# Licensing Novel Role-Governed Categories: An ERP Analysis

**DOI:** 10.3389/fnhum.2015.00633

**Published:** 2015-12-15

**Authors:** Micah B. Goldwater, Arthur B. Markman, Logan T. Trujillo, David M. Schnyer

**Affiliations:** ^1^University of SydneySydney, NSW, Australia; ^2^University of Texas at AustinAustin, TX, USA; ^3^Texas State UniversitySan Marcos, TX, USA

**Keywords:** relational categories, role-governed categories, ERPs (event-related potentials), concept formation, conceptual representation

## Abstract

Markman and Stilwell (2001) argued that many natural categories name roles in relational systems, and so they are *role-governed categories*. This view predicts instantiating a novel relational structure licenses the creation of novel role-governed categories. This paper supports this claim and helps to specify the mechanisms underlying this licensing. Event-related potentials were recorded while participants read passages of text. Participants instantiated novel relational representations by interpreting novel verbs derived from nouns during reading. Sentences later, comprehension of novel role terms derived from the novel verb was facilitated relative to a control condition where the novel verb was paraphrased using the root noun in its familiar form. This comprehension facilitation was marked by a reduced negativity elicited from the role term in the Novel Verb condition relative to the Paraphrase from 400 to 500 ms post-stimulus-onset. This relative difference in negativity is consistent with both the N400, which is a marker of semantic integration, and the Nref effect, which reflects the working memory load required to resolve reference. Additionally, because this increased negativity persisted until 670 ms post-stimulus-onset, and not that the Paraphrase condition elicited an increased positivity (i.e., the P600), we ruled out that the licensing effect is rooted in morphosyntactic processes.

## Introduction

**FIGURE 1:**
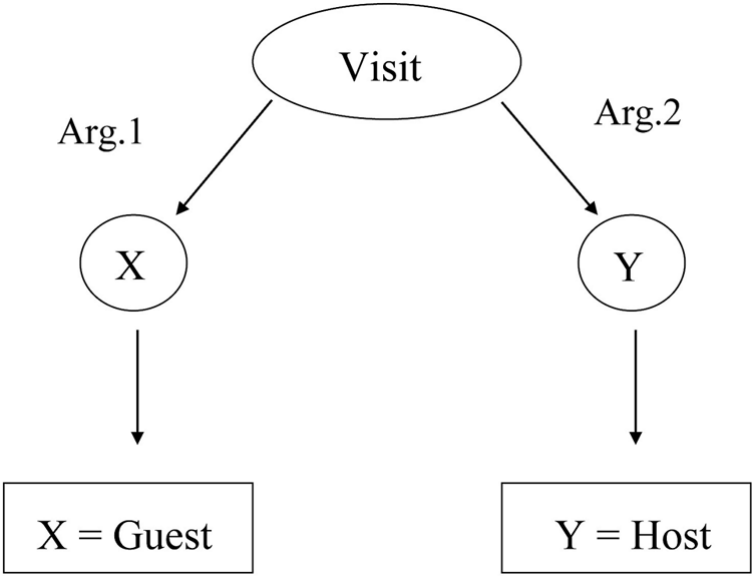
**Representing “visiting” as a relational structure with bound role-governed categories**.

Theories of categorization typically assume that categories are represented by some set of features that describe the properties of category members (e.g., Rosch, 1973). That is, theories assumed that people most often form *feature-based categories*. However, recent work has argued that many natural categories are represented by the roles category members play in situations. For example, *guest* and *host* name different roles in the event of *visiting* (Markman and Stilwell, 2001; Goldwater et al., 2011). Events themselves are represented as relational structures, which are composed of relational predicates and the arguments they bind (see **Figure 1**; Markman, 1999). The concept of *visiting* is relational because it relates two objects: one that is doing the visiting (the guest) and one that is being visited (the host). Categories that name bound relational roles (such as *guest* or *host*) are *role-governed categories* (see Ross and Murphy, 1999; Gentner and Kurtz, 2005; Rein et al., 2010; Goldwater et al., 2011, for empirical demonstrations distinguishing role-governed from feature-based categories).

If role-governed categories name an aspect of a relational structure, then instantiating a novel relational structure should license novel role-governed categories. Goldwater et al. (2011) presented behavioral evidence supporting this hypothesis. However, that research leaves open questions about *how* relational structures license role-govern categories. In this report, we extend these previous findings by recording Event-Related Potentials (ERPs) elicited by Goldwater’s et al. (2011) task to further constrain proposals about the processing mechanisms responsible for the licensing of novel role-governed categories.

### Verbs and Role-governed Categories

The representations of verbs and role-governed categories are intimately connected because verbs are the primary linguistic medium for encoding complex relations. Their representation is one of the most well studied topics in linguistic theory (e.g., Jackendoff, 1990). Verbs point outward to other concepts, specifically to the entities they bind to their argument slots. They bind their arguments by specifying how their arguments relate, for example, through causal action. Each argument has a thematic role in the relation such as the agent (the doer of the action) or the patient (the recipient of the action).

It seems that every role-governed category noun has a corresponding verb or verb phrase. For example, the concept of a *thief* relies crucially on verbs like *steal*, because the defining characteristic of a thief is that this individual is the first argument to the relation × *steals* y. **Table 1** (modified from Goldwater et al., 2011) lists more corresponding verbs and role-governed categories. In these examples, the role-governed categories are distinct lexical items from the verb. English also has the morpheme -*er* that can be used to derive a term that refers to an agent from the verb (e.g., *dance* and *dancer*).

**Table 1:** Verbs and corresponding role governed categories.

Verbs	Role governed categories
x steals y	x = thief
x visits y	x = guest, y = host
x trains/advises y	x = mentor, y = protégé
x gives birth to y	x = mother, y = son or daughter
x defeats y	x = victor/winner, y = loser
x eats y	y = food
x plays y	y = game
x writes y	x = author
x gives y to z	y = gift
x shops in y	x = customer
x lives in y	y = home
x hunts y	x = predator, y = prey

Consistent with this view, McRae et al. (1997) have argued that thematic roles are verb specific concepts rather than abstract roles of *agent* and *patient*. For example, *arrest* assigns arguments to *arrester* and *arrestee* roles (McRae et al., 1997; Ferretti et al., 2001). Ferretti et al. (2001) provide evidence for this view by showing that verbs activate conceptual information about their role-fillers. Verbs prime their typical agents (e.g., *arrest* primes *cop*), their typical patients (e.g., *arrest* primes *criminal*), typical instruments (e.g., *stir* primes *spoon*), and features of patients (e.g., *manipulate* primes *naïve*).

Goldwater et al. (2011) provided further evidence for the strong representational link between verbs and role-governed categories. They demonstrated that learning novel verbs licensed novel role-governed categories. Goldwater et al. (2011) did not claim that learning the novel verbs led to instantiating the role-governed categories themselves, but rather that by creating a relational structure with role-filler slots, later instantiation of novel role concepts is facilitated.

The novel verbs Goldwater et al. (2011) used were derived from nouns. Many familiar verbs are *denominal*, e.g., *dust*, *shelve*, *saddle*, *google*, etc. (see Clark and Clark, 1979 for a comprehensive taxonomy). In addition, novel denominal verbs are readily understood in their first encounter in on-line sentence comprehension (Goldwater and Markman, 2009, see Kaschak and Glenberg, 2000 for an off-line comprehension task) suggesting they are a reasonable object of study for the instantiation of novel semantic representations.

The use of novel denominal verbs also allows the instantiation of relational representations that are rooted in pre-existing knowledge, allowing concept learning to be rapid and to be embedded in the simple reading of short passages. For example, consider this passage from Goldwater et al.’s (2011) materials (italics added for clarity): “Max was running late for a date, and had just come from the gym. Quickly, he *cologned* himself fresh. He knew he had no time for a shower. Max hoped his solution would be enough. Later that evening, the *cologner* felt confident enough to kiss his lady friend.” Goldwater et al. (2011) tested whether these lexical innovations (e.g., “*cologned* himself fresh”) licensed novel role-governed categories by using the -*er* morpheme to create novel agent terms (e.g., referring to Max as the *cologne*). Because relational structures license role-governed categories, interpreting a novel denominal verb rapidly allows one to understand the role-governed category.

To show the link between verbs and role-governed categories, Goldwater et al. (2011) contrasted the use of a novel denominal verb with a paraphrase using the root noun, for example, “Quickly, he used *cologne* to make himself smell fresh.” Besides the use of the novel verb or the paraphrase the passages were identical. The last sentences of the passages, containing the agent term, were read a word (or two) at a time, to enable the measurement of self-paced reading time. The licensing effect is revealed by faster processing of the novel role-governed category, e.g., *the cologner*, when preceded by the novel verb than when preceded by the paraphrase.

In addition, Goldwater et al. (2011) included a second control condition that presented novel adjectives derived from the same set of nouns using the morpheme “-ful,” e.g., *cologneful*. This control condition enabled Goldwater et al. (2011) to show that the licensing effect was specific to the link between verbs and role-governed categories, and not between any two novel terms derived from the same root. That is, because the Novel Verb condition showed the same advantage relative to the Novel Adjective as to the Paraphrase, the licensing effect cannot be due to some sort of general novelty advantage wherein novelty prepares you for more novelty.

While Goldwater et al. (2011) showed faster processing times for learning novel role-governed categories when a novel relational structure is previously instantiated reading time only measures general processing load, it does not necessarily reveal the kind of processing being measured. The goal of this paper is to shed further light on the processes underlying the licensing effect.

We consider three kinds of processes to explain how role-governed categories are licensed: semantic integration processes, reference resolution processes, and morpho-syntactic processes. The rationale for each is explained in turn.

The first possibility is that the licensing effect reflects ease of integration of the novel role concept into a semantic representation of the sentence/discourse. We know that novel category labels are interpreted rapidly on-line in context. Perhaps the same semantic integration processes involved in the construction of a rich representation of a discourse composed entirely of familiar words is also responsible for interpreting novel concepts encountered during discourse construction (Rapp and Gerrig, 1999). The priming results of Ferretti et al. (2001) suggest that the link between verbs and their specific thematic roles are represented in semantic memory. Perhaps forming new representations of verbs and verb-specific thematic roles involves these same processes. That is, the slower processing of the agent term in the Paraphrase condition of Goldwater et al. (2011) could reflect more difficulty updating the discourse representation with the meaning of the novel term, similar to when semantically anomalous or unlikely words are encountered (when compared to more felicitous and predictable words, e.g., Kutas and Hillyard, 1984). This view is consistent with Markman and Stilwell’s (2001) theoretical argument that role-governed categories are a regular and prominent part of semantic memory, and Goldwater et al.’s (2011) interpretation that licensing role-governed categories is a productive process of the conceptual system.

A second possibility is that the licensing effect is reflective of reference resolution processes. When encountering a term that co-refers with a word introduced earlier in the discourse, the comprehender must actively link the new term with its co-referent in memory/the discourse representation (e.g., Chang, 1980; Garrod and Sanford, 1982). For example, this would be needed after first referring to someone as “my long-time companion, Dr. Jones,” and then later referring to her as “my friend.” This process of establishing co-reference relies on working memory, as shown by Daneman and Carpenter’s (1980) findings (among others) that individual differences in working-memory capacity predict reference resolution accuracy. Under this account, the licensing effect reflects a reduction in the working-memory demands to resolve reference, similar to how it is easier to resolve co-reference of unambiguous as compared to ambiguous referring terms (e.g., Van Berkum et al., 2007).

This working-memory reduction account is consistent with Goldwater et al.’s (2011) claims that the licensing reflects the eased binding of a role concept to an already built relational structure. Constructing relational representations and binding relational roles is a working memory intensive process (Waltz et al., 2000). When presented with a role-concept to bind, having that relational structure (with open role-slots) already constructed is potentially a less working memory intensive process than having to construct the relational representation and then bind the role.^1^ In sum, this account posits the licensing effect is rooted in the reduced working memory demands to comprehend the novel role term and link it to its antecedent.

A third possibility is that the licensing effect is rooted in morphological and syntactic processes, and not based in meaning or conceptual representations. On this view, the critical aspect of interpreting a novel usage of the *-er* morpheme is that *-er* is a derivational morpheme that operates over verbs to generate novel nouns. In this case, the agent term is processed faster in the novel verb condition than in the paraphrase condition (and in the novel adjective condition) because there was a root verb from which the *-er* morpheme could be used to derive a noun. That is, in this explanation, it was not important that there was any semantic representation established by the novel verb. The novel verb only eased processing of the novel agent term because the comprehender established a new member of a syntactic category that allowed for this morphological derivation. Evidence for this explanation would fail to provide evidence for the conceptual connection between role-governed categories and relations proposed by Markman and Stilwell (2001).

Reading time measures cannot tease these different explanations apart, because each predicts that the novel verb condition will lead to faster processing of the novel agent term than will the paraphrase condition. However, different components of ERP waveforms correspond to different processes. Before presenting the ERP extension of Goldwater et al.’s (2011) study, we briefly discuss how ERP waveforms mark cognitive processes.

### ERP Components as Processing Measures

Research on linguistic and memory processes has found that components of ERP waveforms are reliable markers of different processes (e.g., Osterhout et al., 2004; Voss and Paller, 2006). That is, differences in amplitude of different waveform components elicited between experimental conditions mark differences in different kinds of processes. Here, we discuss three ERP components and corresponding processes.

A relative difference in negativity between approximately 400 ms post-stimulus-onset (the N400 effect) is a marker of semantic access and integration processes. Typically this effect has a locus in posterior sites, but it can be generated from a variety of brain regions (Kutas and Federmeier, 2011). In sentence processing, a reduced N400 is a sign of relative processing ease to update the semantic representation of the sentence with a new word (which involves both accessing the new word’s meaning and integrating it into representation of the sentence on-line, Kutas and Federmeier, 2011). For example, the size of the N400 decreases with the cloze probability of a word in a sentence (Kutas and Hillyard, 1984).

There are two important kinds of N400 findings relevant to the current work, that each concern how prior discourse context affect online semantic interpretation. (1) The ease of semantic integration that the N400 reflects shows sensitivity to how discourse context constrains the interpretation of individual words, for example by making an otherwise semantic anomaly (e.g., an inanimate object having emotions) seem felicitous (Nieuwland and Van Berkum, 2006; Nieuwland, 2013). (2) After a novel word’s meaning is inferred even from a single usage earlier in a discourse, it can elicit an increased N400 on its next usage if now in a semantically anomalous context (compared to a more felicitous second use Borovsky et al., 2012).

A distinct component with a similar time signature to the N400 reflects ease of referential resolution processes. The Nref effect shows an increased negativity also initiating approximately 400 ms post-stimulus-onset indicating a greater difficulty in resolving reference. Unlike the N400, which is typically measured in posterior sites, the Nref effect has a frontal locus, and this relative negativity often persists into later time epochs (e.g., up to 1600 ms post-stimulus onset Van Berkum et al., 2007). Van Berkum et al. (2007) showed that comprehending a noun that had two potential co-referents introduced earlier in the discourse elicited this increased frontal negativity compared to comprehending a noun with only a single possible co-referent. To explain this effect, they point to the similarity with the timing and spatial distribution of ERP effects of working-memory load (e.g., see Rugg and Allan, 2000), which is consistent with the evidence that reference resolution is working memory dependent (Daneman and Carpenter, 1980).

A later component, typically elicited approximately 600 ms post-stimulus-onset reflects different kinds of processes. In language comprehension, an increased positivity with this timing is associated with detecting morpho-syntactic violations such as number agreement errors, as in *the children runs* (the P600 effect see, e.g., Hagoort et al., 1993; Osterhout et al., 2004).

From these three lines of research we can generate predictions for ERPs elicited from novel role-governed categories. Goldwater et al. (2011) provided evidence that novel relational structures instantiated through the interpretation of novel verbs facilitated the comprehension of novel role terms. We can expand on these findings by comparing ERPs elicited from these novel words. In the present experiment, we used materials from Goldwater et al. (2011). Across conditions, the same novel words (e.g., *cologner*) are used in the same sentences. The only difference between the conditions is whether the root word (e.g., *cologne*) is introduced in its familiar noun form, or as a novel denominal verb three sentences earlier.^2^ Clearly, any difference between waveforms elicited by the same set of novel terms is rooted entirely in how the novel verb facilitates their comprehension.

If licensing novel role-governed categories is driven by semantic updating processes, then the agent term should show a relative decrease in parietal negativity from 400 ms post-stimulus-onset when it was preceded by a novel verb, as compared to the paraphrase. If this licensing effect is driven by working-memory dependent reference resolution, then we would expect the novel verb condition to elicit a reduced frontal negativity initiating 400 ms post-stimulus onset, then persisting for upward of entire additional second. On the other hand, if the novel verb advantage reflects a morpho-syntactic process (i.e., the appropriate use of the derivational morpheme *-er*), and that the agent term in the paraphrase condition is processed as a misuse of derivational morphology, then the difference in waveforms should not appear until later, approximately 600 ms post-stimulus-onset, and in the opposite polarity.^3^ That is, a P600 would entail that the paraphrase condition elicited an increased positivity, not an increased negativity as with either the N400 or Nref effects.

Our account predicts that licensing novel role concepts uses the same semantic and working memory processes that underlie constructing a discourse representation of entirely familiar words. That is, we posit the licensing has a meaningful and conceptual nature, and is not purely based in derivational morpho-syntactic processes. Thus, we predict that the licensing effect will be revealed by an increased negativity elicited by the Paraphrase condition during the earlier time window. However, we are making no *a priori* prediction about whether this negativity will reflect the N400 or Nref.

## Materials and Methods

### Subjects

Twenty-three neurologically normal right-handed native English speakers from the University of Texas participated in the experiment for course-credit. This study was carried out in accordance with the recommendations of the Institutional Review Board of University of Texas at Austin (IRB # 2005-03-0057). All subjects gave written informed consent in accordance with the Declaration of Helsinki.

### Materials

The subjects read a total of 126 (54 critical, 72 filler) five-sentence passages. Of the 54 critical passages, 27 had novel verbs, 27 had paraphrases. All had novel agent terms. Two lists were created such that each critical passage appeared in both conditions across subjects. 27 of the critical passages were used in Goldwater et al. (2011). The number was doubled for the present experiment due to need for a large set of items in language ERP research. See Appendix for representative examples of critical passages.

To ensure subjects paid attention, they judged the sensicality of every sentence in every passage. All sentences in the critical passages were sensical. In the filler passages, one third of the passages had zero non-sense sentences, one third had one non-sense sentence, and one third had two non-sense sentences.

Sentences were either presented all at once, or one word at a time. Having some sentences presented in entirety cut down on the overall length of the experiment, and we also hoped this variation would help the participants keep focus during critical sentences. All sentences with novel words were presented one at a time to allow for ERP recording. Within the filler passages, one third of the passages had zero sentences presented one word at a time, one third presented had one sentence presented one word at a time, and one third had two sentences presented one word at a time.

### Procedure

Subjects sat in a dimly lit room in a comfortable chair in front of a computer monitor, with a mouse at their right hand. They were told that we were interested in the brain’s responses to reading. They would read a series of passages, some of which contained sentences that didn’t make sense. At the end of every sentence, they judged whether it made sense by pressing one mouse button for “makes sense” and another for “doesn’t make sense.” They were informed that some sentences would be presented as a whole, while others would be presented one word at a time. During sentences presented one word at a time, they were to fixate their eyes on the center of the screen until the sentence was complete. The experiment was broken up into four blocks. In between each block, they could rest for as long as they wanted.

Each new passage began with the words “New Passage” presented on the screen for 1500 ms. Before the presentation of every sentence, a fixation cross appeared in the center of the screen for 500 ms. Sentences presented as wholes remained on screen for 3000 ms. During sentences presented one word at a time, each word appeared in the center of the screen for 300 ms, with 400 ms of blank screen in between each word. Appropriate punctuation accompanied the sentence-final word. At the end of every sentence, a row of X’s appeared as a prompt for the sensicality judgment. After the judgment, the screen was blank for 1500 ms before the next fixation cross appeared.

### EEG Recording and Pre-processing

Continuous EEG was recorded using active Ag/AgCl electrodes mounted in a 64-channel BioSemi electrode cap (BioSemi B. V., Amsterdam, The Netherlands; www.biosemi.com) plus two additional sites located on the mastoids. Recording sites in the cap included standard and extended 10–20 system locations. Two additional electrodes were used to monitor electrooculargraphic (EOG) activity (e.g., eye movements and blinks), one at the left inferior orbit and another at the left outer canthi. All channels were amplified by a Biosemi Active II amplifier system in 24 bit DC mode at an initial sampling rate of 2048 Hz (400 Hz bandwidth) decimated online to 256 Hz, with EEG signals recorded with respect to a common mode sense (CMS) active electrode placed between sites PO3 and POZ. As active electrodes make skin preparation redundant, electrode impedances were not measured; however, half-cell potentials of the electrode/gel/skin interface were kept between ±40 mV, following standard recommendations for the Active II system.

Continuous data were imported oﬄine into the MATLAB computing software environment (The Math Works, Inc., Natick, MA, USA) using the EEGLAB toolbox (Delorme and Makeig, 2004) for MATLAB, where all subsequent analysis was performed via in-house scripts that utilized EEGLAB functions. Single 2000 ms EEG epochs were extracted from -750 ms to 1250 ms with respect to the word onset. Next, EEG trials were transformed to a mastoids reference for the purposes of removing muscle and signal artifacts from the EEG record by visual inspection. Blink and saccade-related EOG activity was estimated by computing oﬄine an EOG channel formed from the bipolar montage of the electrodes located at the outer can this and inferior orbit. EEG trials containing EOG amplitudes higher than 50 μV or lower than -50 μV (after removal of the constant direct current offset from the EOG signal) within the -200 pre-stimulus to 800 ms post-stimulus interval were rejected from the analysis in MATLAB via automatic algorithm. An average of 22 ± 0.5 trials per condition was retained after artifact rejection.

After elimination of EOG artifacts, the derived horizontal and vertical EOG channels were removed from the data and the single EEG trials were then transformed to a digitally linked mastoids reference and band-pass filtered between 0.1 and 30 Hz (166 point zero phase shift FIR filter with 0.03 and 5 HZ transition bands, respectively). Epochs were then truncated to -100 ms preceding to 700 ms following word onsets, when the next word was presented. All ERPs were baseline corrected to the -100 to 0 ms pre-stimulus interval.

### Statistical Analysis of ERPs

The statistical identification of the temporal and spatial span of between-condition ERP differences was assessed via pointwise non-parametric randomized permutation paired *t*-tests corrected for multiple comparisons across time and scalp electrode location using a previously published cluster-based method (Nichols and Holmes, 2002; Trujillo et al., 2010; Sanguinetti et al., 2015). The advantage of this statistical procedure is that it does not require *a priori* (and possibly biased) choices of scalp regions and time intervals, while simultaneously providing balanced control over Type I and Type II error. This procedure consisted of three steps. First, we computed statistical significance thresholds (*p* < 0.05 uncorrected) independently for each electrode and time point by estimating a t-distribution from 20,000 random within-subject permutations of data values across conditions under the null hypothesis. These significance thresholds formed a three-dimensional statistical image where the first two dimensions preserved the two-dimensional topographic organization of the electrodes and the third dimension was time. Second, another round of 20,000 permutations computed the null distribution of maximal contiguous 3-D (electrode × electrode × time) cluster exceedance masses, defined as “the integral of the statistic image above the primary threshold within the suprathreshold cluster” (Nichols and Holmes, 2002, p. 8). Here, the primary thresholds were the significance thresholds determined in the first step. Third, observed 3-D cluster exceedance masses were compared to the empirically derived null cluster exceedance mass distribution; those clusters with exceedance masses greater than the *p* = 0.05 criterion were significant at an adjusted two-tailed level. Permutation testing was applied simultaneously all time points and electrodes (except the mastoid reference sites). We remapped the final 3-D statistical maps into two dimensions for ease of visualization.

## Results

Participants were accurate on 82% of the total number of trials, properly judging the sensicality of presented sentences. Furthermore, for the critical sentences that presented the novel verbs (99% sensical), paraphrases (98% sensical), and novel role-governed category terms (100% sensical for both conditions) participants showed ceiling levels of accuracy.

**FIGURE 2:**
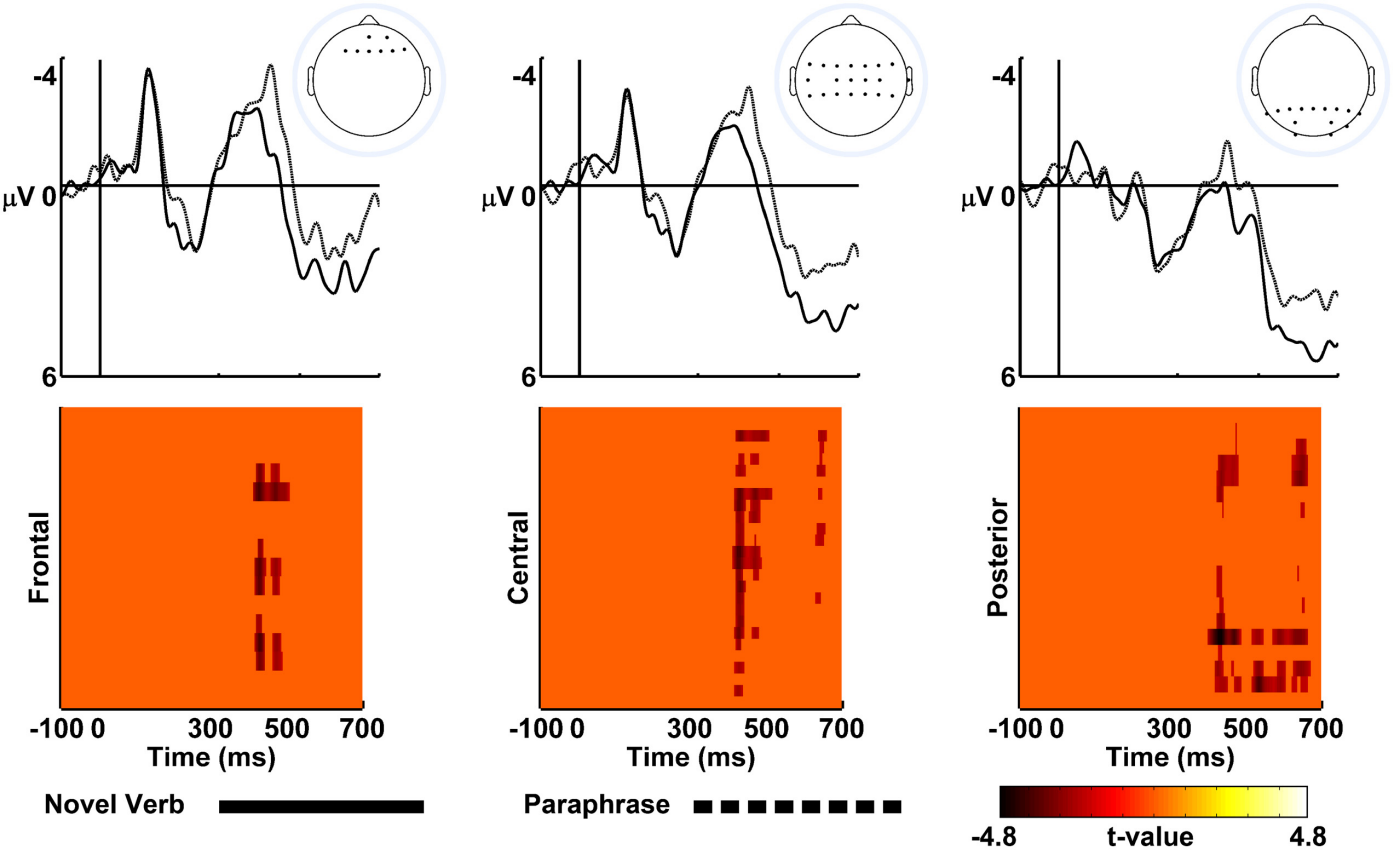
**Event-related potential (ERP) effects of role-governed category licensing. (Top)** Show ERPs elicited by the novel agent terms in the novel verb (solid line) and paraphrase (dashed line) conditions recorded at frontal scalp sites (left column), central scalp sites (middle column), and posterior scalp sites (right column). The onsets of agent terms are indicated by the vertical black lines; negative voltage is plotted up. The ERPs are averaged across electrodes demonstrating statistically significant between-condition differences; top panel head map insets show the scalp locations (black dots) of electrodes entering into each ERP average. **(Bottom)** Show corresponding statistical cluster maps comparing the Paraphrase versus Novel Verb conditions; light/dark colors indicate +/- *t*-values.

**FIGURE 3:**
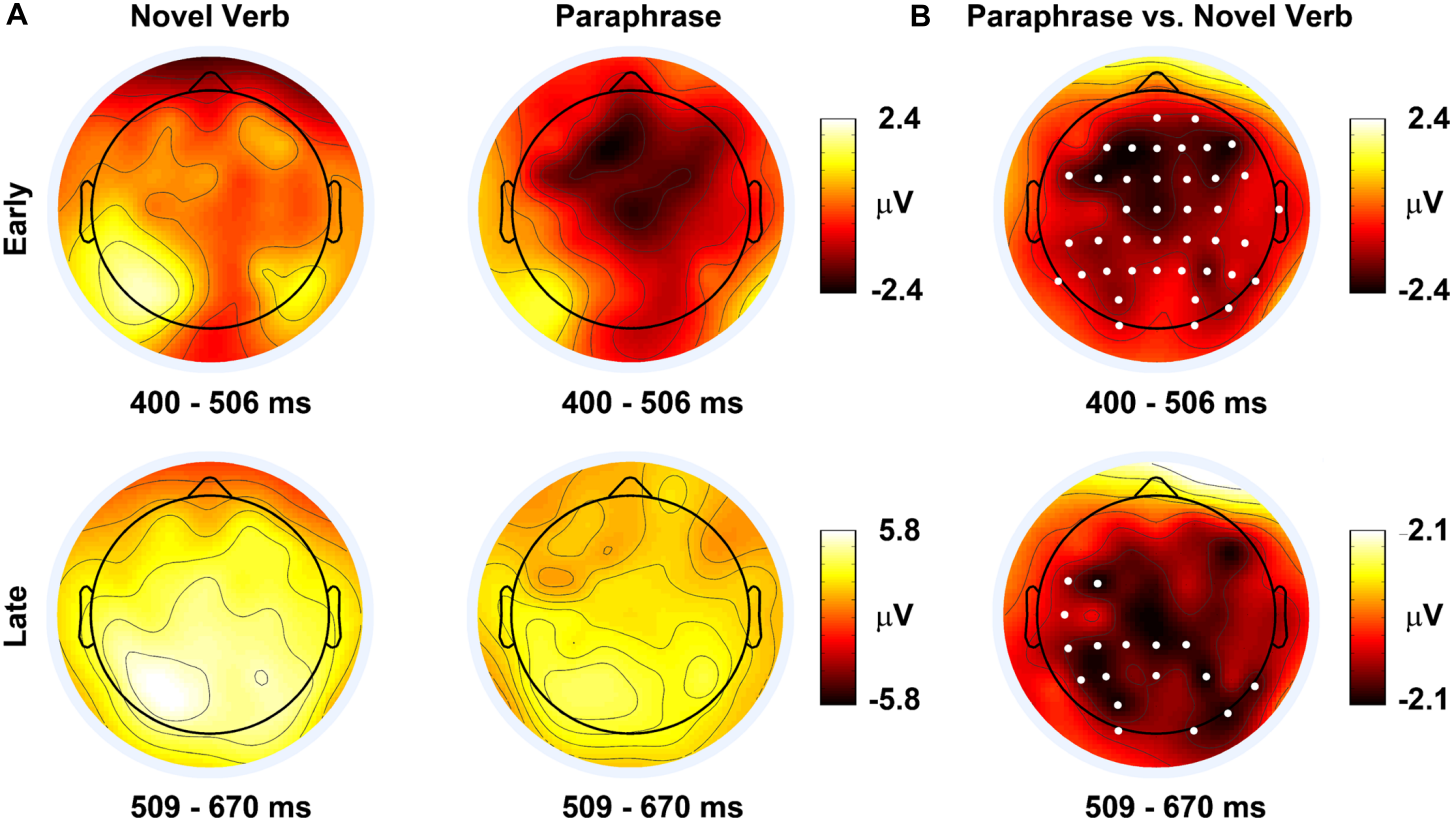
**(A)** Event-related potential scalp topographies and **(B)** ERP difference topographies averaged over time periods of significant between-condition differences. The noses of the headmaps point upward. The white dots on the difference maps indicate electrodes where the permutation cluster analysis revealed statistically significant differences (see Materials and Methods, *p* < 0.05, two-tailed, 20,000 permutations).

The ERP’s were time locked to the onset of the novel role-governed category term, e.g., *cologner*. The ERP waveforms (**Figure 2**) exhibited a negative polarity response over frontocentral locations from 300 to 500 ms and a positive polarity response over posterior scalp locations between 500 and 700 ms. The ERP scalp topographies within these time periods were consistent with the topographies of the N400/Nref and P600 components, respectively (**Figure 3A**). Statistical testing showed that the ERPs were more negative in the Paraphrase versus Novel Verb conditions over medial frontocentral sites and parietal sites from 400 to 506 ms (within the time period of the N400/Nref topography) and widespread posterior sites from 509 to 670 ms (within the time period of the P600 topography); see **Figures 2** and **3**.

This pattern of data leads to some strong conclusions about the nature of the licensing effect, but also leaves some ambiguity. First, there is a clear difference between the two conditions. Second, there was no evidence that the differences were due to interpreting the novel agent-term in the paraphrase condition as an error in derivational morphology. The Paraphrase condition, which would have elicited an increased *positivity* if it represented a morphosyntactic violation, maintained its increased *negativity* from the previous time period. However, it is not entirely clear whether the differences in negativity (from the earlier time period) more closely reflect the N400 or Nref effects. On the one hand, the locus of the early effect was more anterior to many N400 effects in language comprehension, suggesting an Nref effect. On the other hand, there still was statistically reliable differences in posterior cites in the early time window, quite consistent with the N400. Perhaps more importantly, it was these posterior differences that persisted into the later time window and not the frontal differences. Frontal sites often show the Nref effect for 1500 ms post-stimulus onset (Nieuwland and Van Berkum, 2008), but in the current data, the frontal sites did not show a statistically significant difference between conditions after 500 ms post-stimulus onset (though qualitatively, the difference remained). Overall, this pattern does not appear entirely conclusive, but on the whole seems more consistent with a N400, rather than an Nref effect.

## Discussion

Goldwater et al. (2011) found that instantiating a relational structure via novel denominal verb interpretation licensed novel role-governed categories. The goal of the present study was to reveal what underlying neural mechanisms were responsible for this licensing effect. We recorded ERPs because different components of the ERP waveform often reflect different processes. Changes in the ERP waveform 400 ms post-stimulus-onset have been shown to reflect semantic integration (e.g., Kutas and Hillyard, 1984) or reference resolution (Van Berkum et al., 2007). While, differences 600 ms post-stimulus-onset have been shown to reflect morphosynactic processes (e.g., Hagoort et al., 1993; Osterhout et al., 2004). We observed that the agent term in the novel verb condition elicited a reduced negativity from 400 to 506 ms as compared with the paraphrase condition, broadly consistent with an N400 or Nref effect (Kutas and Hillyard, 1984; Van Berkum et al., 2007). Further, there was suggestive evidence that this effect had a frontocentral locus. This increased negativity persisted to 670 ms in the posterior sites, which was in the reverse direction of what a morphosyntactic violation would elicit. Taken together, we are confident in ruling out a morphosyntactic account of the licensing effect, and cautiously suggest that the effect is most consistent with the N400.

These findings are important because they suggest that licensing novel role concepts during language comprehension involves the same kinds of semantic, and perhaps working memory, and referential resolution processes as is used when comprehending discourse made of entirely familiar words. Novel role-concepts are readily incorporated into a semantic representation of a discourse when they are licensed by a verb. This provides important support for the framework of category representation arguing for the prominence of categories defined by the roles in relational structures laid out by Markman and Stilwell (2001) and Goldwater et al. (2011). If role-governed categories are as prevalent as proposed in this framework, then they must be straightforward to acquire (i.e., when given the proper linguistic or conceptual support). This account is consistent with both Nref and N400 explanations. If the former, it suggests that the licensing effect is rooted in reducing the working-memory demands of comprehending the novel role term and linking it to its co-referent. If the latter, it suggests that the licensing effect is rooted in easing the processes required to update a semantic representation with the meaning of the novel role term.

It was also important that there was no P600 effect. A relatively uninteresting interpretation of the licensing effect obtained by Goldwater et al. (2011) is that it is merely due to rules of morphological derivation. If the advantage for the agent term in the novel verb condition were rooted in a grammatical derivation of nouns from verbs devoid of meaning, then the difference would not have been revealed in components reflecting semantic or referential processing (see Kim and Osterhout, 2005 for similar discussion). Indeed, the paraphrase even elicited a *less* positive-going waveform, during the later time window than the novel verb condition, which is in the opposite direction of an effect caused by morpho-syntactic error detection (Hagoort et al., 1993).

The current findings are also relevant for the language processing literature more generally. Models of the lexicon typically represent a fixed set of meanings (c.f. Pustejovsky, 1991), however, often language comprehension, as seen here, entails creating new meanings. Clark and Gerrig (1983) criticized standard lexicon models because they are only capable of “sense selection” and not “sense creation.” Consistent with Rapp and Gerrig (1999), this paper suggests that understanding novel words in language comprehension, at least when rooted in existing semantic knowledge, likely engages the same system that is used to understand familiar words (and see Borovsky et al., 2012). Likewise, with just a bit of supporting context (e.g., a counter-factual framing), semantic processes are flexible enough to readily interpret otherwise anomalous meanings as sensible (Nieuwland and Van Berkum, 2006; Nieuwland, 2013). While models of the lexicon need to be augmented to account for such results, these findings suggest that many of the same mechanisms are at work.

This work focuses on role-governed category acquisition embedded in discourse comprehension. Our general claim is that relational structures license role-governed categories. There are no doubt other contexts where role-governed concepts are formed that rely on processes beyond typical discourse comprehension. For example, gaining expertise in science can be seen as a problem in relational categorization (Chi et al., 1981; Rottman et al., 2012). If our framework is correct, then part of gaining such expertise will be forming novel role-governed categories as part of these relational representations. Future work will investigate these processes.

The goal of this paper was to better explain how instantiating a novel relational structure licenses novel role-governed categories. When this licensing occurs in the context of discourse comprehension, semantic, referential, and working memory processes (but not morpho-syntactic derivation) appear responsible for the effect. This is critical support for the framework of category representation laid out in Markman and Stilwell (2001) and Goldwater et al. (2011) because it shows that role concepts engage similar systems as the rest of our concepts, suggesting any account of concepts and categories that leaves them out, is far from complete.

## Conflict of Interest Statement

The authors declare that the research was conducted in the absence of any commercial or financial relationships that could be construed as a potential conflict of interest.

## Footnotes

1 However, it should be noted that models of relational representation differ in that some view relational structures as entirely the binding of roles (e.g., Hummel and Holyoak, 1997), while others view relations and role representations as distinct (e.g., Falkenhainer et al., 1989). Though, it is the former model that emphasizes the role of working memory in role-binding.

2 Because the “novelty” explanation was ruled out in the previous study with the Novel Adjective condition, we only used the single control condition to reduce overall length of the experiment.

3 While typically the P600 has been elicited by errors with inflectional morphemes (such as verb tense or number agreement), it seems reasonable that such effects could extend to errors in derivational morphology (as in the improper use of the *-er* morpheme).
