# Supplementary material for: Licensing Novel Role-Governed Categories: An ERP Analysis
Source: Front Hum Neurosci. 2015 Dec 15;9:633. doi: 10.3389/fnhum.2015.00633 (PMC4678187; doi:10.3389/fnhum.2015.00633)
Supplement: Supplementary file 1 [file Data_Sheet_1.PDF]

Appendix

Sample Passages

Cologne

Novel Verb

Max was running late for a date, and had just come from the gym. Quickly, he cologned himself fresh. He knew he had no time for a shower. Max hoped his solution would be enough. Later that evening, the cologner felt confident enough to kiss his lady friend.

Paraphrase

Max was running late for a date, and had just come from the gym. Quickly, he used cologne to make himself smell fresh. He knew he had no time for a shower. Max hoped his solution would be enough. Later that evening, the cologner felt confident enough to kiss his lady friend.

Hairpin

Novel Verb

Tricia realized she couldn't get into her apartment. She hairpinned the lock open. Her keys were nowhere to be found. Tricia is a resourceful woman. After getting inside, the hairpinner found her spare key to put in her purse.

Paraphrase

Tricia realized she couldn't get into her apartment. She used her hairpin to jiggle the lock open. Her keys were nowhere to be found. Tricia is a resourceful woman. After getting inside, the hairpinner found her spare key to put in her purse.

Lead-pipe

Novel Verb

Ralph and Sal were fixing their plumbing together. Sal got pretty annoyed and lead-piped Ralph's knee. They were trash talking each other for a while now. Someone was bound to burst. After a couple of seconds, the lead-piper found a bandage to stop the bleeding.

Paraphrase

Ralph and Sal were fixing their plumbing together. Sal got pretty annoyed and used a lead-pipe to hit Ralph's knee. They were trash talking each other for a while now. Someone was bound to burst. After a couple of seconds, the lead-piper found a bandage to stop the bleeding.

Vine

Novel Verb

Walter bought a new house, but wanted it to look older. He decided to vine the porch and terrace. He had considered changing the siding. However, Walter really likes plants. For the next project, the viner added a hottub to spruce up the bathroom.

Paraphrase

Walter bought a new house, but wanted it to look older. He decided to use vines to decorate the porch and terrace. He had considered changing the siding. However, Walter really likes plants. For the next project, the viner added a hottub to spruce up the bathroom.

Candle

Novel Verb

Marshall was relaxing at home went the lights when out. He candled the room brighter. It was his first night off in a while and he wanted to read his novel. Marshall was annoyed that another fuse had blown. After a few minutes, the candler sat down in his chair to return to his book.

Paraphrase

Marshall was relaxing at home when the lights went out. He used a candle to make the room brighter. It was his first night off in a while and he wanted to read his novel. Marshall was annoyed that another fuse had blown. After a few minutes, the candler sat down in his chair to return to his book.
